# Dental Workload Reduction during First SARS-CoV-2/COVID-19 Lockdown in Germany: A Cross-Sectional Survey

**DOI:** 10.3390/ijerph18063164

**Published:** 2021-03-19

**Authors:** Thomas Gerhard Wolf, James Deschner, Harald Schrader, Peter Bührens, Gudrun Kaps-Richter, Maria Grazia Cagetti, Guglielmo Campus

**Affiliations:** 1 Department of Restorative, Preventive and Pediatric Dentistry, School of Dental Medicine, University of Bern, CH-3010 Bern, Switzerland; guglielmo.campus@zmk.unibe.ch; 2 Department of Periodontology and Operative Dentistry, University Medical Center of the Johannes Gutenberg-University Mainz, D-55131 Mainz, Germany; james.deschner@uni-mainz.de; 3 Free Association of German Dentists/Freier Verband Deutscher Zahnärzte (FVDZ), D-53117 Bonn, Germany; has.FVDZ@fvdz.de (H.S.); drs.buehrens@t-online.de (P.B.); gudrun.kaps@t-online.de (G.K.-R.); 4 Department of Biomedical, Surgical and Dental Science, University of Milan, I-20142 Milan, Italy; maria.cagetti@unimi.it; 5 Department of Surgery, Microsurgery and Medicine Sciences, School of Dentistry, University of Sassari, I-07100 Sassari, Italy; 6 WHO Collaborating Centre for Epidemiology and Community Dentistry, University of Milan, I-20142 Milan, Italy

**Keywords:** COVID-19, dental practice, economic, dentist, Germany, global pandemic, reduction, SARS-CoV-2, workload

## Abstract

An observational cross-sectional survey was planned to analyze the weekly workload reduction of German dentists during lockdown due to the global COVID-19 pandemic. Participants were predominantly members of the Free Association of German Dentists and filled in an online questionnaire. The questionnaire was sent to a total of 9416 dentists, with a response rate of 27.98% (*n* = 2635). Respondents were divided into seven macro areas by gross domestic product. Nearly two-thirds of dentists (65.16%) reported a reduction in their practice workload of more than 50% compared to the pre-pandemic period with statistically significant differences between German macro areas (*p* < 0.01). Weekly workload was reduced during the lockdown in 93.00% of study participants, while 55.33% dental care centers with multiple employed dentists under the direction of a non-dentist general manager had only a 40% reduction in weekly workload compared to a solo practice or a practice of a dentist with an employed dentist (30.24% and 28.39%, respectively). Dentists in Germany drastically reduced their practice activity during the first wave of the COVID-19 lockdown, both in rural and urban areas. Short, medium, and long-term effects of the pandemic on dental practices, dental staff as well as patient care need to be further investigated.

## 1. Introduction

The coronavirus SARS-CoV-2 (severe acute respiratory syndrome coronavirus 2) pandemic has altered the world deeply. COVID-19, as the disease has become known, is the third coronavirus to emerge in the human population recently. In Germany, the first confirmed case COVID-19 pandemic has been recorded since the end of January 2020 according to the Ministry of Health in Bavaria (Southern Germany, and since then, new cases have been reported continuously, and patients have increasingly been isolated in hospitals [1]. On 27 March, the German federal parliament (Deutscher Bundestag) passed a law entitled “Law to protect the population in the event of an epidemic situation of national importance”. This allows orders to be issued at the federal level in the federal health system without the consent of the Federal Council (Bundesrat) [2]. Since then, a worldwide travel warning has been issued, non-essential travel to the EU has been restricted, numerous shops have been closed, and an entry ban for third-country nationals has been implemented as a measure (lockdown). Contact restrictions have been in place since 22 March [1]. A two-week domestic quarantine obligation for returnees from abroad at the beginning of April was followed by a mask obligation on public transport and in shops in mid- to late April. Since then, the obligation to wear protective masks on public transport and when shopping has been in force in all federal states. The distance and hygiene measures have changed regularly since then, depending on the specific federal state, and various events and even district regions have been quarantined. There have been regular adjustments as well as loosening, which are reviewed and re-evaluated daily by politicians with regard to the R-value and daily infection figures by the Robert Koch Institute in Berlin (Germany) [1,3].

Dental services were also deeply affected by the pandemic and a general reduction of working hours/week and during lockdown dental treatments were generally suspended, except for emergency care [4,5,6]. Regarding dental services, during the lockdown, many regions in Germany as in other countries [7] restricted dental care to emergencies [8].

The National Association of Statutory Health Insurance Dentists (Kassenzahnärztliche Bundesvereinigung (KZBV)) and the German Federal states’ dental chamber (Bundeszahnärztekammer (BZÄK)), together with the regional dental authorities (Kassenzahnärztliche Vereinigungen der Länder (KZV)), have developed a joint package of measures to maintain the provision of dental care in Germany even in times of increasing spread of SARS-CoV-2/COVID-19. Thus, all measures at the federal and state level should be coordinated and harmonized. In addition, a synchronized level of information should be ensured and uncertainty among patients and dentists avoided. In addition to recommendations for ensuring dental care in compliance with infection control, the package of measures of the dental profession includes, among other things, a proposal for the provision of acute dental emergency treatment for infected and quarantined patients in specialized practices and treatment centers in hospitals. For reasons of infection control and maintenance of dental care for the breadth of the population, treatment of infected or quarantined patients in practices should be avoided as much as possible. Emergency care for infected and quarantined patients should be organized through specially designated clinics as dental treatment centers. This measure should serve to prevent the spread of the virus as far as possible in the course of dental treatment, to reduce the risk of infection for patients and practice staff, and to ensure the long-term security of care. In addition to these treatment centers, the regional dental authorities have designated special focus practices in the states for the emergency treatment of infected or quarantined patients. In terms of contractual dental care, § 95, 3, 1 of the German Social Code, Book V (Sozialgesetzbuch, SGB V) stipulates that contractual dentists are entitled and obligated by virtue of their license to participate in contractual dental care to the extent of the care mandate resulting from their license. Exceptions to this basic obligation to treat can only come about through officially ordered practice closures in accordance with the Infection Protection Act. In exceptional cases with justified special features, a temporary practice closure can also take place in coordination with the responsible KZV or the local health authority [9].

The University of Bern designed and carried out a global survey to evaluate the impact of the COVID-19 outbreak among dentists working in different countries [10]. In Germany, the survey was accomplished in collaboration with the Free Association of German Dentists (Freier Verband Deutscher Zahnärzte (FVDZ)), the biggest free professional organization of dentists in the Country and the University of Mainz (Germany). Information regarding protective measures and awareness of dentists has been already presented in several European countries [11,12]. With 83.2 million inhabitants (18.6% of the total EU population), Germany is the most populous EU Member State, followed by France (67.1 million; 15.0%), Italy (60.2 million; 13.5%), Spain (47.3 million; 10.6%), and Poland (38.0 million; 8.5%) [13]. The hypothesis behind this investigation was to analyze the factors related to the weekly workload reduction of German dentists apart the COVID-19 pandemic. To verify this hypothesis, an online survey was designed and carried out during June 2020 involving German dentists.

## 2. Materials and Methods

### 2.1. Development and Calibration of the Questionnaire

The questionnaire development was previously described, standardized, and published [11]. It is an anonymous questionnaire divided into four domains. Further information about the questionnaire has been published in previous papers [10,11]. The standardization of the questionnaire is described in detail in the previous paper [10]; briefly, the questionnaire was pre-tested on a small group (*n* = 42); Intraclass Correlation Coefficients (ICC) was run for the test–retest and intra-rater reliability for each item. An ICC value of 0.80 or higher was considered satisfactory. Only two items showed an ICC below the threshold, and after discussion among the authors, the questions were slightly modified.

The questionnaire was built in English and, in order to ensure a correct procedure for cross-cultural adaptation and linguistic validation, a translation/back-translation procedure was designed and followed. The questionnaire was forward translated into German by two translators who are native German that are fluent in English and have experience of the issue. After the translation, a consensus version was identified and subsequently back-translated into English by an independent person who was not involved in the study to guarantee the accuracy and comparability of the translation. The questionnaire was divided in several items. One item comprised age, gender, work status, region, and area of living and practice; another one was related to the dentists’ practice during the COVID-19 period (lockdown, first and second re-opening), others were related to COVID-19 related symptoms referred by participants and protective measures used during their clinical practices. The present paper is focused on the analysis of the first two items.

### 2.2. Online Survey

All dentists (*n* = 9416) recorded in the database of the Free Association of German Dentists (Freier Verband Deutscher Zahnärzte (FVDZ), Bonn, Germany) were contacted by e-mail asking their participation, of which 1902 were employed dentists, 7258 were self-employed dentists, and 256 were orthodontists. Only dentists to accept the survey received the questionnaire. The data were received and recovered in the database of Lime Survey, which is a free online survey application (Version 4.3.14, LimeSurvey GmbH, Hamburg, Germany). The study was performed according to the guidelines of the Declaration of Helsinki of 1964 with its further amendments. It was approved by the Ethics Committee of the Rhineland-Palatinate Medical Association/Landesärztekammer Rheinland-Pfalz (Mainz, Germany) with a positive vote on 9 June 2020 (Reference number 2020-15112). The questionnaire was available for thirteen days (5–17 June 2020).

### 2.3. Data Analysis

All the data obtained from the completed questionnaires were export in a spreadsheet (Excel 2020 for Mac, Microsoft, Redmond, WA, USA), cleaned, and finally transferred in Stata 16 (StataCorp LLC, College Station, TX, USA, http://www.Stata.com, accessed on 8 February 2021) for the statistical analysis. During the period of the survey, the number of COVID-19 cases in Germany were in the last 7 days from 2.6/100,000 (5 June 2020) with 10.4 deaths/100,000 to 2.5/100,000 (17 June 2020) with 10.6 deaths/100,000 [3].

The region and area where participants referred to work was categorized. The 16 German regions were grouped in seven macro areas derived from a 7-State-model as a proposal for the restructuring of the Federal States and the reform of fiscal equalization [14]: Baden-Württemberg (Baden Wurttemberg) (BW), Bayern (Bavaria) (BY), Nordrhein Westfalen (North Rhine Westphalia) (NW), Berlin, Brandenburg, Mecklenburg-Vorpommern (Mecklenburg-West Pomerania) (BE-BB-MV), Bremen, Hamburg, Niedersachsen (Lower Saxony), Schleswig-Holstein (HB-HH-NI-SH), Hessen, Rheinland-Pfalz (Rhineland Palatinate), Saarland (HE-RP-SL), Sachsen (Saxony), Sachsen-Anhalt (Saxony Anhalt), and Thüringen (SN-ST-TH). The Gross National Product of each macro area was used as a proxy of the economic situation of each area [15].

The area of practice was categorized into rural town (2000–5000 inhabitants), small town (5000–20,000 inhabitants), medium town (20,000–100,000 inhabitants), and large town (more than 100,000 inhabitants). Participants’ work status was organized as *a*—employed in a private practice; *b*—owner of a private practice; *c*—private practice/state healthcare; *d*—state healthcare dentist. The type of practice was coded as single practice, practice with employed dentist/s, medical/dental care centers with several employed dentists under the direction of a non-dentist managing director, and university/public health. The percentage of weekly workload, assuming that the weekly working time for a full-time is 42 h, during the different phases of the pandemic was also recorded.

Absolute and relative frequencies were calculated for each item. Difference in proportion was evaluated with χ^2^ test or Fisher exact test if one cell had a value of less than five. Multiple testing for post hoc estimation, such as the number of observed frequencies, expected frequencies, percentage, and contribution to the chi-square were run. A nonparametric test for trend across ordered different macro areas, workload during the lockdown and the reopening phases, and the German macro areas GNP was appraised. Principal Component Analyses (PCA) were used for exploratory data analysis and for making predictive models, and the principal components (eigenvectors of the data’s covariance matrix) were plotted in an orthogonal graph. PCA is a statistical procedure that converts a set of observations of possibly correlated variables into a set of values of uncorrelated variables, called principal components, using an orthogonal transformation. Quite often, PCA operation can be thought of as revealing the internal structure of the data to explain in the best way the variance in the data. PCA is closely related to factor analysis; indeed, some statistical packages (such as Stata) deliberately conflate the two techniques. Comparing to traditional regression analysis, PCA is used for estimating the unknown regression coefficients in a standard linear regression model. All the variables (Gender, GNP/German macro areas, areas of practice, reduction of workload, type of practice) were imputed in the PCA analysis as explanatory variables. A value of *p* less than 0.05 was considered statistically significant.

## 3. Results

**Figure 1:**
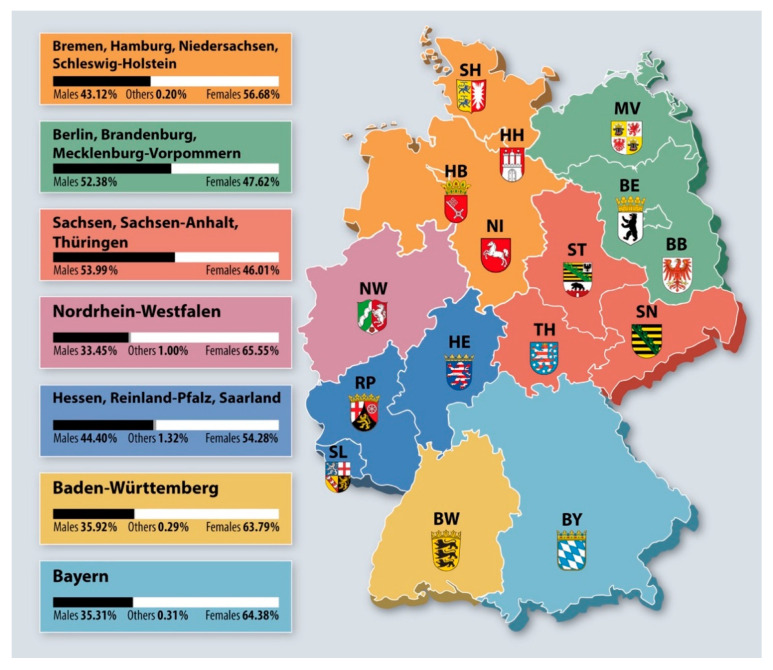
Distribution of German states into macro-areas and gender among study participants (*n* = 2635).

A total of 2635 dentists participated and concluded the survey, i.e., 27.98% of the all the dentists recorded in the database of Free Association of German Dentists (FVDZ). The distribution of responders by macro areas ranged between 5.58% (HB-HH-NI-SH) and 22.58% (HE-RP-SL); more than 4/5 (87.07%) of the dentists participating in the survey are dentists who own their own practice. Table 1 displays the distribution of the responders by gender and region of working. A statistically significant difference among gender and macro areas (Figure 1) was observed (χ^2^_(12)_ = 62.40, *p* < 0.01). The sample was harmoniously distributed by gender and work area (χ^2^_(6)_ = 2.87, *p* = 0.83). During the lockdown period (after discussion and decision-making by the German Chancellor with Prime Ministers of the federal states on 22 March 2020 [16]), almost two-thirds of dentists (65.16%) reported a reduction of clinic activity of more than 50% with respect to the period before the pandemic with a statistically significant difference among the German macro areas (χ^2^_(18)_ = 55.80, *p* < 0.01) (data not in tables).

The percentage of weekly workload during the lockdown was reduced in 93.00% of the responders, while no statistically significant association was observed (χ^2^_(24)_ = 29.83, *p* = 0.19) among macro areas (Table 2), a statistically significant nonparametric test for trend across workload and macro areas GNP was detected (z = 4.73, *p* = 0.01).

The highest percentage of reduction (81–100%) was quite similar among the different German macro areas, while the lowest percentage of reduction was statically significant different among the macro areas, 18.12% in BW vs. 10.24% in SN-ST-TH (χ^2^_(6)_ = 14.91, *p* = 0.03). The reduction of the weekly workload during the lockdown (Table 3) was not statistically significant associated to the number of inhabitants of the areas where the dentists practiced the profession (χ^2^_(12)_ = 8.51, *p* = 0.75).

The type of practice, where the dentists declared to work was statistically significant associated to the percentage of the workload reduction during the lockdown period (χ^2^_(12)_ = 24.08, *p* = 0.02); in particular, more than 50% (55.33%) of those responders who affirmed to work in medical/dental care centers with several employed dentists under the direction of a non-dentist managing director have a reduction of only 40% of the weekly workload compared those working in a single practice or in practice with an employed dentist/s (30.24% and 28.39%, respectively) (Table 4).

**Figure 2:**
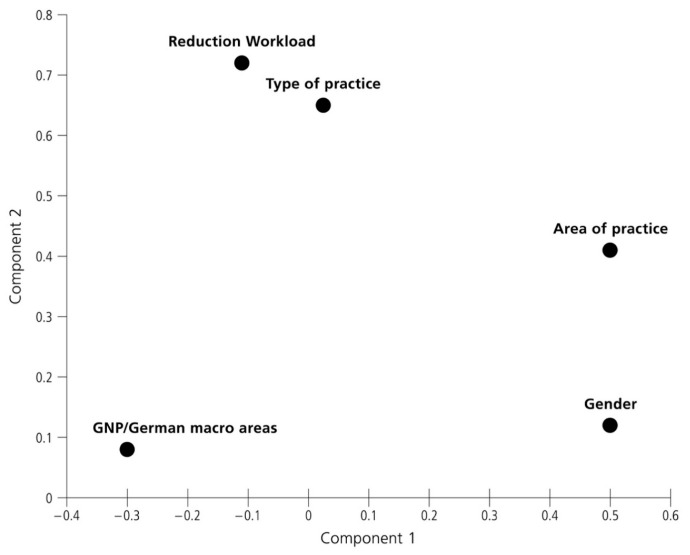
Orthogonal Rotation (varimax) of the first two principal coordinates in the total sample, the percentage of reduction of the weekly workload during the lockdown period, and the type of practice tend to form a separate cluster with a high goodness of fit (65.64%).

Principal Component Analysis (PCA) was performed on the dataset and the first two eigenvalues, obtained from distance matrix between groups, collectively account for more than 66% of the total variance (68.18%). Figure 2 displays the Orthogonal Rotation (varimax) of the first two principal coordinates in the total sample, the percentage of reduction of the weekly workload during the lockdown period and the type of practice tend to form a separate cluster with a high goodness of fit (65.64%); the GNP of the different macro areas, gender, and the area of practice were clearly separated from the other variables. The weekly workload reduction after the first relaxation measure, which gradually allowed the first opening measures after 20 April, was again statistically significant associated with the type of practice (χ^2^_(12)_ = 30.34, *p* < 0.01) with the responders who affirmed to work in medical/dental care centers with the majority of employed dentists under the direction of a non-dentist managing director (83.25%) reported to have resuming the pre-COVID workload period (data not in table).

## 4. Discussion

The present survey was designed and conducted during the waning of the first wave of COVID-19 in Germany. Study participants indicated that the weekly workload was drastically reduced during the lockdown, both in rural and urban areas. A statistically significant nonparametric test for trend across ordered groups was observed between workload and macro areas in terms of gross domestic product during low workload. The reduction in weekly workload during the lockdown was not statistically significantly associated with the population of the areas of dental practice activity. However, the type of practice was statistically significantly associated with the percentage of workload reduction during the lockdown.

### 4.1. Burdening Effects

The COVID-19 pandemic has hampered an impressive psychological stress on mankind, in particular the medical and dental workforce [17]. Intensive work as a health care provider is both physically and emotionally stressful [18,19,20], even though dentists do not work night shifts, they are more likely than the general population to develop depression, anxiety, somatization, or insomnia. Relevant risk factors are daily working hours, high BMI, and females, whereas age, family income, and years of working are protective factors for psychological disorders [20]. Regular intensive training for effective crisis management is required because, despite resilience and professional dedication, the intense work is physically and emotionally demanding [18]. Dental personnel are at high risk for respiratory infectious diseases and characterized by a high tolerance to prolonged stress [21]. For example, dental personnel working directly on the front lines are more than four times more likely than the general population to be affected by anxiety disorders [22]. Furthermore, exposure to potentially infectious agents, performance of aerosol-generated services, the workload, and job performance are not significantly associated with anxiety. However, older age and protective measures such as medical uniform, gown, medical cap, N95 respirator, goggles, face shield, medical hazmat suit, gloves, and medical shoe covers [23] seem to decrease the anxiety level of FDS, while conflicts with colleagues and/or patients worsen the anxiety level [21].

### 4.2. Economic Impact

Dental care spending was recognized to be in decline by 38% in 2020 and 20% in 2021 [24], without having including in the modeling analysis the second COVID-19 wave, as we are observing. Unemployment ratio and economic disruption are rising rapidly; more than 16 million people are expected to lose their employer-sponsored dental insurance in the United States of America (USA) [25]. This circumstance will cause an immediate reduction on the supply of dental care: a decrease in routine checkups, an increase in tooth extractions, and more dental emergencies [25]. A statistically significant decrease in weekly scheduled patients was observed in public with respect to private clinics; thus, increasing disparities due to differences in care between public and private networks will be expected [26]. Male dentists reported being significantly more affected by economic losses than female dentists, which subsequently led to the application for government assistance. Public health dentists had more emergency patients per week than private dentists. However, not only did the number of patients treated decrease, but the level of care was also reported as impaired [27]. While all dental services were affected by reduction, prevention, periodontics, and prosthodontics were the most affected, with reductions of nearly 20% for public and private services [28]. To buffer these effects, economic subsidies may be necessary to avoid both short- and medium-term cash shortages. Gradual relaxation of the measures imposed by the government can be expected to lead to a sharp increase in the need for dental treatment in dental practices. This could be accompanied by an increase in medical emergencies, which must be specifically addressed with regard to the risk of COVID-19 transmission [29].

### 4.3. Limitations

Some limitations must be highlighted. First, the response rate was quite low given the high number of questionnaires sent compared to the total number of dentists (70,740) dentists in Germany [30]; however, the sample size reached might be considered an inference of the dental population in Germany as generalizing from observations made on a sample to a larger population [31]. Secondly, due to the constantly changing situation in the different German states during the COVID-19 pandemic, the dentists’ perceptions during the outbreak and lockdown changed significantly over the summer, and the results of this survey may not adequately reflect the current situation as the COVID-19 regulations and the constantly changing different measures. One of the main outcomes of the survey was an association between the workload during the reopening period and the type of practice, even if the number and type (predominantly independent practice) of participating dentists might be not representative of all German dental population.

However, the data of the present survey are of extreme importance for political decision-makers, for the estimation of the reduction in working hours and consequences. Although SARS-CoV-2 and COVID-19 are still not fully researched and understood, there is a great challenge among health professionals in general, which is accompanied by economic effects, triggered e.g., by positive infections within the dental team, which can be transmitted either privately or occupationally by aerosol formation, missing patient flows due to appointment cancellations or postponements due to fear as a patient of a risk group.

## 5. Conclusions

Weekly workload was drastically reduced during the lockdown in Germany in both rural and urban areas. A statistically significant trend between workload and macro areas in terms of gross domestic product at low workload was observed. Small practice structures, such as dental offices with only one practitioner or an additional employed dentist, reduced their workload significantly more than non-dentist-managed dental centers with numerous practitioners. Rehabilitation of the practice to pre-pandemic conditions after the end of the lockdown also occurred more rapidly in larger practice structures than in small ones. As the pandemic situation is evolving, very sudden, short-, medium-, and long-term economic effects of the pandemic on dental practices, dental staff, and patient care need to be closely monitored with new surveys.

## Figures and Tables

**Table 1:** Responders’ distribution among gender and German macro areas. As the number of respondents (gender = other) was practically nil, only males and females were considered for the statistical analysis.

	**GNP Ranking of German Macro Areas**							
**Gender**	**BY** ***n* (%)**	**BW** ***n* (%)**	**HB-HH-NI-SH** ***n* (%)**	**HE-RP-SL ** ***n* (%)**	**NW** ***n* (%)**	**BE-BB-MV ** ***n* (%)**	**SN-ST-TH ** ***n* (%)**	**Total** ***n* (%)**
Males	113 (35.31)	125 (35.92)	213 (43.12)	202 (44.40)	199 (33.45)	77 (52.38)	149 (53.99)	1078 (40.91)
Females	206 (64.38)	222 (63.79)	280 (56.68)	247 (54.29)	390 (65.55)	70 (47.62)	127 (46.01)	1542 (58.52)
Other	1 (0.31)	1 (0.29)	1 (0.20)	6 (1.32)	6 (1.01)	--	--	15 (0.57)
Total	320 (12.14)	348 (13.21)	494 (20.89)	455 (17.27)	595 (22.58)	147 (5.79)	276 (10.47)	2635

χ^2^_(12)_ = 62.40, *p* < 0.01.

**Table 2:** Weekly workload reduction during the lockdown period among the German macro areas ranked by Gross National Product (GNP).

Workload	GNP Ranking of German Macro Areas							
% Reduction	BY *n* (%)	BW *n* (%)	HB-HH-NI-SH *n* (%)	HE-RP-SL *n* (%)	NW *n* (%)	BE-BB-MV *n* (%)	SN-ST-TH *n* (%)	Total *n* (%)
<20%	70 (14.74)	25 (18.12)	33 (12.84)	55 (12.67)	62 (11.29)	36 (10.98)	30 (10.24)	311 (12.57)
21–40%	110 (23.16)	24 (17.39)	48 (16.68)	81 (18.66)	101 (18.40)	59 (17.99)	52 (17.75)	475 (19.20)
41–50%	128 (26.95)	37 (26.81)	62 (24.12)	120 (27.65)	164 (29.87)	113 (34.45)	95 (32.42)	719 (29.06)
61–80%	100 (21.05)	33 (23.91)	70 (27.24)	121 (27.88)	142 (25.87)	83 (25.30)	73 (24.91)	622 (25.14)
81–100%	67 (14.11)	19 (13.77)	44 (17.12)	57 (13.13)	80 (14.57)	37 (11.28)	43 (14.68)	347 (14.03)
Total	475 (20.00)	138 (5.58)	257 (10.38)	434 (17.54)	549 (22.20)	328 (13.26)	293 (11.84)	2474

No responders = 161 (6.11%), χ^2^_(24__)_ = 29.83, *p* = 0.19.

**Table 3:** Weekly workload reduction during the lockdown period by in the different practice areas grouped by number of inhabitants.

Workload	Area of Practice				
% Reduction	Rural Town *n* (%)	Small Town *n* (%)	Medium Town *n* (%)	Large Town *n* (%)	Total *n* (%)
<20%	101 (12.55)	88 (13.92)	71 (11.02)	51 (13.04)	311 (12.58)
21–40%	141 (17.52)	24 (18.83)	142 (22.05)	72 (18.41)	474 (19.17)
41–50%	249 (30.93)	174 (27.53)	180 (27.95)	113 (28.90)	716 (28.96)
61–80%	199 (24.72)	164 (25.95)	161 (25.00)	102 (26.09)	626 (25.33)
81–100%	115 (14.29)	87 (13.77)	90 (13.98)	53 (13.55)	345 (13.96)
Total	805 (37.56)	632 (25.57)	644 (26.05)	391 (15.82)	2472

No responders = 163 (6.18%), χ^2^_(12__)_ = 8.51, *p* = 0.75.

**Table 4:** Weekly workload reduction during the lockdown period by the type practice where the responders declared to work.

Workload	Type of Practice				
% Reduction	Single Practice *n* (%)	Practice with Employed Dentist *n* (%)	Medical/Dental Care Centers *n* (%)	University/Public Health *n* (%)	Total *n* (%)
<20%	190 (11.61)	46 (8.11)	81 (33.06)	13 (19.12)	330 (13.14)
21–40%	305 (18.63)	115 (20.28)	57 (23.27)	14 (20.59)	491 (19.55)
41–50%	507 (30.97)	168 (29.63)	28 (11.43)	12 (19.12)	716 (28.50)
61–80%	418 (25.53)	157 (27.69)	44 (17.96)	16 (23.53)	635 (25.28)
81–100%	217 (12.52)	81 (14.29)	30 (12.24)	12 (17.65)	345 (13.53)
Total	1637 (65.17)	567 (22.57)	240 (9.55)	68 (2.71)	2517

No responders = 118 (4.48), χ^2^_(12__)_ = 24.08, *p* = 0.02.

## Data Availability

The data presented in this study are available on request from the corresponding author. The data are not publicly available due to the General European Data Protection Regulation (GDPR) of 25 May 2018.
